# Pediatric Rattlesnake Envenomation: A Simulation Scenario With Optional Health Equity, Virtual Facilitation, and Senior Learner Modifications

**DOI:** 10.7759/cureus.18106

**Published:** 2021-09-19

**Authors:** Melissa N Joseph, Elizabeth Sanseau, Anita Thomas, Nick Brandehoff, Julie Augenstein, Megan Shuster, Gunjan Tiyyagura, Marc Auerbach

**Affiliations:** 1 Emergency Medicine, Yale University, New Haven, USA; 2 Pediatric Emergency Medicine, Children's Hospital of Philadelphia, Philadelphia, USA; 3 Pediatrics, Seattle Children's Hospital, Seattle, USA; 4 Emergency Medicine, University of Colorado, Aurora, USA; 5 Pediatric Emergency Medicine, Phoenix Children's Hospital, Phoenix, USA; 6 Pharmacology, Yale New Haven Hospital, New Haven, USA; 7 Pediatric Emergency Medicine, Yale University, New Haven, USA

**Keywords:** toxicology and envenomation, social determinants of health (sdoh), virtual simulations, antivenom, uninsured patients, allergy and anaphylaxis, high fidelity simulation, parental refusal of care

## Abstract

Rattlesnake envenomation is an uncommon but urgent cause of presentation for emergency care. Recognition of envenomation, timely administration of antivenom when indicated, and recognition of antivenom reactions are of critical importance to mitigate the local, hematologic, and systemic effects of Crotaline venom. This technical report describes the presentation and use of a simulation-based scenario of an envenomated child who requires treatment with antivenom. Optional additions to this scenario are described and include antivenom reaction, health equity considerations, and virtual facilitation.

## Introduction

Pit viper envenomation is a low frequency, yet high stakes cause for presentation to the emergency department (ED) for both adult and pediatric patients [1]. Rattlesnake is a member of the Crotalinae subfamily of pit vipers. ED personnel must recognize the signs and symptoms of potential envenomation and treat appropriately to avoid significant injury or death. The treatment of pit viper envenomation can also have significant adverse reactions that clinicians should be able to identify and treat. 

Pit vipers account for approximately 9,000 bites per year and three to five deaths per year in the United States (US). Up to 25% of bites can be "dry bites," where no envenomation occurs. Of those who are envenomated, symptoms can range from minor, local reactions to systemic syndromes including severe hypotension, confusion, seizures, respiratory failure, paralysis, rhabdomyolysis, and coagulopathies leading to serious bleeding [1].

Timely recognition and treatment of envenomation are imperative for clinical outcomes of the patient with pit viper envenomation. Identification of fang marks is unreliable, and physicians should maintain a high index of suspicion for snake envenomation in the adult or child who presents with sudden onset of pain in an appropriate clinical context.

Currently, there are two antivenoms available that can be utilized for the treatment of pit viper envenomation: Crotalidae polyvalent immune fab (ovine), CroFab® (Btg International Inc., Conshohocken, US), and Crotalidae immune F(ab′)2 (equine), ANAVIP® (Rare Disease Therapeutics, Inc., Franklin, US). Both of these medications are indicated for adult and pediatric patients who develop any signs of envenomation after sustaining a bite wound from a suspected pit viper in the US. The dose is the same for adult and pediatric patients without adjustment for age or weight; the goal is to bind the amount of venom introduced by the snake bite [2-5].

Pit viper antivenom is costly. CroFab and ANAVIP cost approximately US$3400 and US$1200 per vial wholesale, respectively, and the starting dose for CroFab is four to six vials and 10 vials for ANAVIP. Additionally, the charge per vial by the hospital to the patient may be much greater and patients who are envenomated and receive antivenom should be admitted to a monitored setting. This presents the possibility of a significant cost of treatment, which can be particularly impactful in the uninsured or underinsured patient.

In this technical report, we present a simulated pediatric patient who presents with envenomation by a rattlesnake and requires antivenom administration as well as consideration of the cost of care and navigating associated parental concerns.

## Technical report

Methods

The design of this simulation incorporated medical management of crotalid envenomation with social aspects of caregiver concerns regarding cost of treatment. This scenario was facilitated both in-person as well as virtually and was adapted to both junior learners, defined as resident physicians with little prior exposure to envenomations, and senior learners, which were pediatric emergency medicine (PEM) fellows in our case. It was run both with and without an active observer and critical action checklist. At the conclusion of the scenario, facilitators allowed for debriefing through self-reflection and guided action review as well as a didactic component. Where the health equity supplement was used, a "pause and restart" technique was used to debrief the interaction and resources available before moving on to the remainder of the medical management of the case.

The simulation was developed to help pediatric and general emergency medicine physicians recognize and treat a child with crotalid envenomation. Through participation in this simulation, learners have the opportunity to practice communication with the patient and caregiver, address parental concerns regarding cost, practice obtaining and delivering antivenom, and recognize and treat antivenom reactions (Table 1). Though it was originally developed for residents and fellows, it may also be helpful for attending physicians and other members of the healthcare team such as emergency department nursing staff as a means to review a rare but important emergent presentation.

**Table 1:** Learning Objectives

Learning objectives (adapted from the American Board of Pediatrics)	
Junior Learner	Recognize the signs and symptoms of life- and limb-threatening complications of snake envenomations.
	Execute the management of snake envenomation by type and severity.
	Identify which snake envenomations are liable to produce significant illness or injury in children.
	Implement an appropriate disposition for the child bitten by a snake.
	Recall the available antivenoms for pit viper envenomation and their indications, administration, and potential reactions.
Senior Learner	Recognize anaphylaxis secondary to antivenom administration.
	Execute strategies to treat and stabilize the patient as well as continue with antivenom administration.
	Use appropriate communication with the patient, family, and consultants.
Health Equity	Use communication strategies when a parent wants to leave against medical advice.
	Identify the concern related to health equity/lack of insurance.
	Recall the process by which parental refusal for treatment can be overruled.
	Summarize resources available for uninsured/underinsured patient populations

Personnel

Instructor: Bedside nurse can provide details of physical examination where appropriate. If additional instructors are available, one may observe and utilize the active observer checklist for use during debriefing.

Parent: Additional instructor or standardized actor to play the role of the young parent. If additional personnel available, facilitators can also have someone to play the grandparent.

Patient:May be voiced by an instructor or responses may be relayed through the parent or nursing role.

Social Worker:If electing to include the health equity supplement, a social worker confederate is helpful, if consulted, to explain options to the parent.

Learners:Ideally two to three learners

Pediatric and/or emergency medicine residents

Pediatric emergency medicine fellows

Wilderness medicine fellows

Toxicology fellows

Learner preparation

Pre-briefing

Prior to the simulation start, learners should be pre-briefed. This should include reviewing the session’s goals and objectives, the establishment of a fiction contract, logistical details of the simulation, and a commitment to respecting the learners [6].

Environmental Preparation/Equipment: The setting is a pediatric or general emergency department treatment room as applicable to the learners. This simulation can be conducted in situ, virtually, or in a simulation lab using either a high-fidelity or low-fidelity mannequin. We used both a virtual resuscitation room (VRR) set-up as well as a high-fidelity mannequin.

In-person simulation: An age-appropriate mannequin should be sitting on a gurney. Erythema moulage can be applied to a limb. Photos of the wound should be prepared to either share via a monitor screen or as a photo printout with the participants. Intravenous (IV) supplies, IV fluids, monitoring equipment should be available. Ideally, a pediatric crash cart would be available to participants, but at a minimum, the following medications should be available:

1. Normal Saline or Lactated Ringers Solution

2. Acetaminophen

3. Morphine

4. Antivenom

5. Epinephrine (IM and IV)

6. Steroids (prednisone, methylprednisolone)

7. Diphenhydramine IM/IV

8. Tetanus toxoid

Vitals signs should be displayed in real time or, with a low-fidelity mannequin, provided verbally or via a simulator application for phone or tablet.

Virtual Simulation

**Figure 1:**
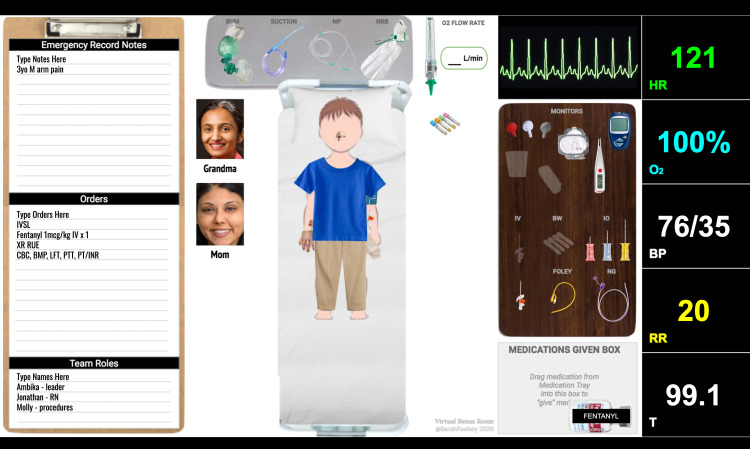
Virtual Resuscitation Room

The authors utilized the VRR developed by Dr. Sarah Foohey and adapted it to this scenario (Figure 1) [7]. The scenario used is open access. Alternatively, facilitators could utilize a virtual meeting platform that allows for screen-sharing and share the images in real time. Facilitators could additionally share real-time vital signs via screen share of their simulation software or web-based application.

Virtual case summary

The patient in our virtual case scenario is Carlos, a three-year-old Hispanic male, brought in by his mother after he came inside from playing around the campsite. He is holding his arm and crying inconsolably (Table 2). Due to his age and distress, he is unable to provide further information regarding what happened. Learners will examine the patient and note vital signs significant for tachycardia, mild hypotension, right arm swelling, and two small puncture wounds to the extremity concerning for a snake envenomation. They should mark the area of swelling, start an IV in the unaffected extremity, check labs and coagulation studies, elevate the arm to 60 degrees, and administer pain control. Once pain control is given, Carlos will confirm that he was bitten by a snake and that his face feels "tingly". He will note that the snake sounded like a "bee buzzing".

**Table 2:** History and Physical Exam HEENT: head, eyes, ears, nose, and throat; HPI: history of present illness; BP: blood pressure; P: pulse; R: respiratory rate

History and Physical Exam	
Triage complaint (provided to learners)	Carlos is a three year old male who is brought in by mom by private vehicle for arm pain. Mom reports that Carlos was playing when he came to her holding his arm and crying inconsolably. She has been unable to get Carlos to explain what happened.
Further history available	Carlos, a three-year-old male, is brought to triage in the emergency department (ED) by his 17-year-old mother at the advice of his grandfather with a swollen right arm. They were camping in the region and he suddenly started to complain of an “owie” in his arm at the campsite this morning (about two hours ago). They noted some blood and tenderness on his palm and assumed this was from a minor fall while playing in the woods while they were packing up at the campsite and put a bandage on the hand. He cried and screamed for a bit but then seemed fine after a bit of consoling. While traveling he started complaining of worsening pain on his right arm and they stopped and got him some medicine. It is now an hour after the Tylenol and ice and he has even more pain, swelling and redness so when grandpa noted the ED sign on the highway he decided to come to get it checked.
Allergies	None known
Medications	None
Past medical/family history	Full term, fully immunized. No hospitalizations. Family history noncontributory.
Social history	Carlos and his mother are from Guatemala. They came to the United States two years ago and are living with Carlos’ grandfather. They do not have insurance.
Review of systems	Pertinent elements as per HPI, other elements are negative.
Physical Examination	
Initial vital signs	BP 83/45, P 115, RR 20, Oxygen saturation: 100%, Temp 99.1
General	Awake, alert, crying, holding arm
HEENT	Normocephalic, atraumatic, pupils equal, round, and reactive to light, no orbital or facial edema or trauma. Oropharynx clear.
Neck	Supple, no lymphadenopathy
Lungs	Clear to auscultation bilateral
Cardiovascular	S1, S2 normal, tachycardic, regular rhythm, no murmurs/rubs/gallops
Abdomen	Soft, no rebound or guarding
Neurological	Moving all extremities spontaneously aside from the right upper extremity, but will pull arm away from examiner with attempts to examine
Skin	Right arm with two small puncture wounds, approximately 20mm apart with surrounding soft tissue edema and oozing from puncture sites
GU	Bilat descended testes with normal lie, normal exam
Psychiatric	Tearful as above, affect congruent to situation

Edema will progress and the learners will need to recognize that antivenom is indicated, order it, and administer in conjunction with toxicology/poison control and a pharmacist. For junior learners, the case ends with disposition to the pediatric ICU (Table 3). For senior learners, the patient goes on to have an anaphylactoid reaction to the antivenom, necessitating treatment with epinephrine, slowed infusion rate, and/or cessation of infusion in conjunction with discussion with poison control (Table 3). There is an option to include a health equity aspect, which in this case we present as parental concern regarding cost of care in the uninsured patient. We recommend approaching this with a "pause and restart" approach, with an initial debrief of the resources available to the uninsured or underinsured patient population as well as strategies to approach the parent who would like to refuse treatment immediately following the simulated discussion. The debrief can be expanded if desired to include other aspects of health equity and obstacles such as poverty and discrimination, lack of access to good jobs with fair pay, quality education, housing, healthcare, and safe environments [8].

**Table 3:** Scenario Parameters, Prompts, and Scripts NSAID: non-steroidal anti-inflammatory drugs; BP: blood pressure; P: pulse; RR: respiratory rate; SaO2: arterial oxygen saturation; T: temperature; VS: vital signs; CBC: complete blood count; IM: intramuscular;

Part 1		
Intervention/Time Point	Expected learner actions	Additional Information/prompts
Case start	Obtain history of present illness, past medical history, past surgical history from parent and grandparent. Assess vital signs and interpret appropriately according to age. Perform a physical examination that must include evaluation of the chest, lungs, mental status, affected extremity, and skin.	Cost concerns will not be immediately uncovered unless specifically asked. Initial vital signs are within normal limits for age. See Table 1 for physical exam findings.
Recognition of possible snake bite. If learners do not recognize the possibility of a snake bite, mom can volunteer that she heard a “weird buzzing sound” at the campsite.	Identify puncture wounds concerning for snake bite (Figure 2). Elevate the extremity and document the degree of edema/erythema upon presentation. Appropriately manage pain (non-NSAID drugs, IV opioids preferred). Order laboratory examinations including CBC, coagulation studies, type and screen, and interpret appropriately. Consider x-ray to evaluate for foreign body, fracture if on differential diagnosis. Communicate effectively with the child’s parent: Exam findings and preliminary diagnosis, plan for initial workup. Answer questions and provide guidance/anticipation as appropriate.	Note that not all wounds have the classic appearance – can be single or multiple punctures or a scratch. Area of edema/circumference measurement should be marked on the extremity. Mom is concerned about the cost of the x-ray and labs. If participants reassure her of the necessity, she will be ok to proceed.
Participants are told 15 minutes has passed. Vital signs: BP 81/43, P 112, RR 20, SaO2 100% T 99.1. Labs are available. Exam: Edema has progressed to the forearm and fasciculations are noted (Video 1).	Learners must: Repeat examination of the affected extremity with serial markings of the leading edge of swelling/redness and circumferential measurements. (Learners can utilize tape or removable sticker dots as an alternative to marking pen if utilizing a mannequin.) Reassess vital signs. Address tetanus status. Recognize laboratory abnormalities and progression of swelling as signs of envenomation and indications for antivenom. Consult toxicology/poison control and pharmacist prior to administering antivenom. Ask and document allergies, ask specifically about prior antivenom exposures or known allergy to sheep, papaya, pineapple (CroFab) or horse protein (AnaVip) [3,4].	For health equity portion, proceed to health equity script after learners verbalize need for antivenom. Otherwise, proceed to Part 2 or Part 3 depending on your learner level. Pharmacist tells team to be careful with the vials due to cost of US$10,000 per vial. They recommend an initial five vials of CroFab or 10 vials of AnaVip. Pharmacy and/or poison control will prompt the allergies if not asked.
Part 2 (Junior Learners – if Senior Learner, skip to part 3)		
If no antivenom administered VS: BP 76/35, P 121, RR 20, SaO2 100%, T 99.1 Exam: Edema has extended to upper arm		Prompt possibility of snake bite from RN or mom.
If correct dose of antivenom is administered, participants are told that 1 hour has passed. VS: BP 87/46, P 111, RR 20, SaO2 100%, T 99.1. Exam: Edema has progressed (1cm increase in circumference at previously marked margin). Participants now note epistaxis on exam.	Learners must: Administer additional antivenom, communicate with parent/patient.	
If repeat dose of antivenom is administered: VS 92/50, P 102, RR 20, SaO2 100%, T 99.1. Exam: Edema is unchanged.	Learners must: Recognize stability of exam. Admit to appropriate level of care (ICU). Communicate findings and plan to parent and family. Recommend updating tetanus prophylaxis as warranted. Recommend against using NSAID drugs for pain control or inflammation (IV opioids preferred).	End case
Part 3 (Senior Learners)		
If no antivenom administered: VS: BP 76/35, P 121, RR 20, SaO2 100%, T 99.1. Exam: Edema has extended to upper arm.		Prompt possibility of snake bite from RN or mom.
If antivenom is administered, participants are told that 10 minutes have passed. VS: BP 72/33, P 135, RR 26, SaO2 94%, T 99.5. Exam: Edema has progressed (1cm increase in circumference at previously marked margin). Patient now with diffuse, urticarial rash, bilateral wheeze.	Learners must: Recognize anaphylaxis. Stop the antivenom infusion. Treat anaphylaxis with epinephrine. Communicate with parent/patient.	Epinephrine IM or IV drip in an appropriate dose and concentration is acceptable.
Epinephrine given . VS: 92/50, P 102, RR 20, SaO2 100%, T 99.1. Exam: Rash, wheeze resolved. Edema progressed 1cm.	Learners must: Recognize need for additional antivenom. Consider restarting after steroids +/- epinephrine drip vs change to alternative antivenom.	Note that AnaVip is FDA approved only for Rattlesnake bites currently.
Antivenom restarted. VS 92/50, P 102, RR 20, SaO2 100%, T 99.1. Exam: Edema is unchanged.	Recognize stability of exam. Admit to appropriate level of care (ICU). Communicate findings and plan to parent and family. Recommend updating tetanus prophylaxis as warranted. Recommend against using NSAID drugs for pain control or inflammation (IV opioids preferred).	End case

After the diagnosis of the bite and recommendation of antivenom, the caregiver will raise concerns regarding the cost of treatment and request to leave. The participants should explore this concern and will discover that the patient is undocumented and the caregiver is still paying for medical bills related to a broken arm she personally had two years ago. Participants should counsel the caregiver regarding the seriousness of the bite and available resources for emergency care and cost assistance (may vary based on site). After the conclusion of this conversation, the facilitator can "pause" the simulation and debrief that aspect and provide site-specific resources. After this specific debrief, the simulation can "restart" and the learners would proceed with administering antivenom and continue through the conclusion of the case and subsequent debriefing and didactics.

Simulation Scenario

**Figure 2:**
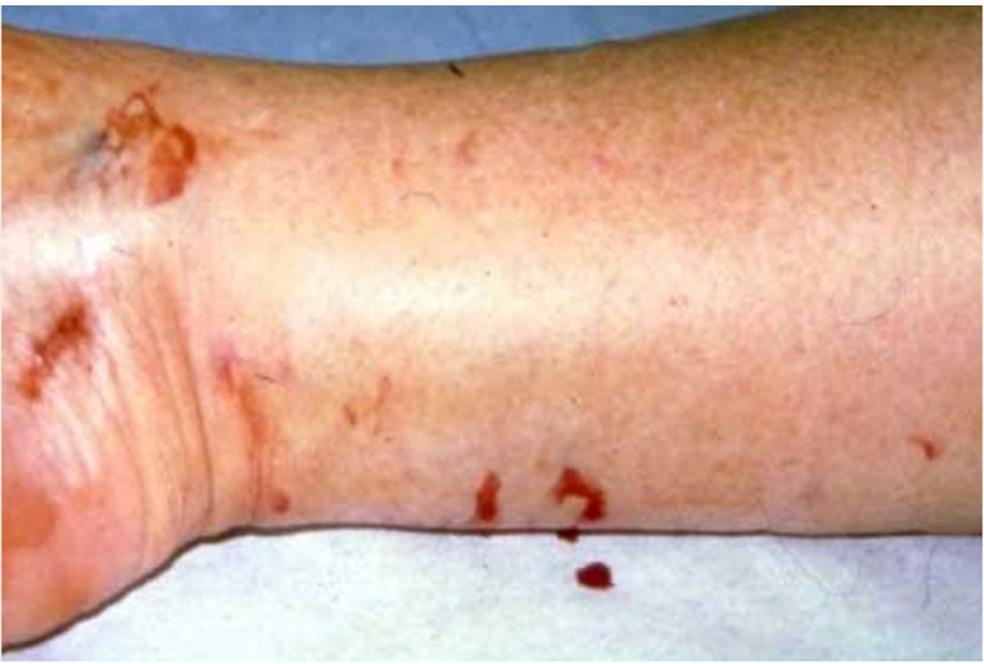
Puncture Wounds Image credit/permission: Greg Ostrow, MD, Virtual Resus Room

Figure 2 and Video 1 are scenario media from the VRR used.

**Video 1:** Fasciculations Video Video media/permission for use: Greg Ostrow, MD, Virtual Resus Room

Optional Inclusion: Health Equity Supplement

The patient triaged with a chief complaint of hand swelling/pain. X-ray ordered and resident marked area of edema and provided ice and ibuprofen. The bedside nurse calls you into the room because mom reports that she wants to leave and that Grandpa made them come to the hospital. When you come to the room you see a swollen/red/bruised hand that is exquisitely tender to palpation and note that Carlos is in severe pain/crying/screaming/kicking.

Context: If asked she is worried about the cost of an x-ray, she is. She and Carlos are undocumented and will not be able to pay for his care. Carlos does not have insurance and they are not supposed to be in the US. Carlos and his mother came to New York City (NYC) two years ago from Guatemala to live with her father (Grandpa) who has been working at a restaurant in NYC for the past 20 years. Mom has a large hospital bill from a broken arm last year that she is still trying to pay off after visiting a clinic in Queens. Mom is reassured that he does need the x-ray and agrees to proceed.

After the patient clinically worsens and participants move to administer antivenom, mom overhears that each vial costs US$10,000. She is more insistent to leave, citing cost concerns and that she read on her phone that she can treat a rattlesnake bite with diphenhydramine. Learners must address mom’s concerns and consider whether she would be allowed to leave against medical advice with Carlos at this point. They should consider utilizing additional resources, such as the clinical social worker and/or patient financial services, who will inform mom that Carlos is eligible for emergency Medicaid. Once appropriately reassured, mom will be amenable to stay. At this point, the simulation should be paused to debrief this social/health equity aspect of the case before proceeding to Part 2 or Part 3.

Process

How The Case Runs From Perspective of Resident

After the pre-briefing, learners are given the chief complaint and have an opportunity to gather history, physical exam, and interpret vital signs. Exam should prompt concern regarding a snake bite and the extremity should be elevated and edema marked. Pain control should be provided and appropriate labwork, including coagulation studies, should be ordered. Upon re-evaluations, learners will note a progression of edema and recognize that antivenom is indicated. At this point, if elected, the patient’s mother may express concerns regarding the cost of treatment and be hesitant to continue treatment. Learners should appropriately address mom’s concerns and offer resources (social work, patient financial services, online resources as available). Learners should also consider whether it would be appropriate to allow this patient to leave against medical advice and what steps would need to be taken if not. Once appropriately counseled, the patient’s mother is agreeable to continuing treatment. In consultation with poison control and clinical pharmacist, the patient is given Crofab or Anavip as per availability. The case ends with updating mom and disposition to the pediatric ICU. For senior learners, the patient has an anaphylactoid reaction to the antivenom. Learners must recognize this, stop the antivenom, and treat with epinephrine. Once stabilized, the infusion can be restarted with epinephrine drip or, if available, the alternative antivenom administered.

Learner Critical Actions Checklist 

Initial evaluation of the bitten child

◻ Obtain history of present illness, past medical history, past surgical history from parent and child

◻ Assess vital signs and interpret appropriately according to age

◻ Perform a physical examination that must include evaluation of the chest, lungs, mental status, affected extremity, and skin.

Envenomation-specific evaluation

◻ Identify puncture wounds concerning for snake bite (note that not all wounds have the classic appearance - can be single or multiple punctures or a scratch)

◻ Elevate the extremity and evaluate the degree of edema / erythema upon presentation

◻ Appropriately manage pain (non-NSAID drugs, IV opioids preferred)

◻ Recognize progression of local tissue response and systemic symptoms as indications for antivenom

◻ Administer CroFab or AnaVip in conjunction with toxicology

◻ Order laboratory examinations including complete blood count (CBC), coagulation studies, type and screen, and interpret appropriately

◻ Admit to higher level of care/pediatric ICU/transfer if needed

◻ Updated tetanus vaccine as indicated

◻ Demonstrate teamwork and appropriate communication within the team

Interpersonal communication

◻ Consult toxicology and demonstrate clear communication and consult question

◻ Communicate effectively with the child’s parent

a. Exam findings and preliminary diagnosis, plan for initial workup

b. Risks, benefits, alternatives of antivenom administration

c. Disposition and rationale

d. Answer questions and provide guidance/anticipation as appropriate

◻ Clear team communication, shared mental model, prioritization of care

Debriefing

Following the conclusion of the simulated case, a debriefing session following simulation debriefing best practices should be facilitated with the learners [9,10]. Relevant educational points are included in Table 4.

**Table 4:** Debriefing Teaching Points DIC: disseminated intravascular coagulation; US: United States; CBC: complete blood count; PT: prothrombin time; PTT: partial thromboplastin time; CK: creatine kinase; UA: urine analysis; NSAID: non-steroidal anti-inflammatory drug; SDU: stepdown unit; CHIP: Children’s Health Insurance Program; ACA: Affordable Care Act

Objective	Discussion points
Recognize the signs and symptoms of life- and limb-threatening complications of snake envenomations	Rattlesnake venom is incredibly complex. It varies even within species, depending on geographic location, time of year, the age of the snake, and other factors. Within venom, there are over 100 documented components that may contribute to symptoms of envenomation. Yet there are several basic syndromes we commonly see: 1. Soft tissue injury: Numerous venom enzymes produce local tissue damage. The damage can be extensive and very painful due to tissue distension combined with the venom effect. Swelling can extend up the extremity as venom travels via the lymphatic system. Most swelling is due to dependent edema. Local bleeding from puncture wounds or in the form of hemorrhagic blebs is common. More diffuse local tissue bleeding is rare. 2. Hematotoxicity: Various venom enzymes can affect the coagulation cascade resulting in decreased clotting times. This can take the form of decreased fibrinogen, elevated D-dimer, and significant thrombocytopenia by causing platelets to adhere to the endothelial wall of blood vessels and this is often referred to as DIC. DIC is incredibly uncommon in the setting of North American snakebite envenomations. Some venoms result in platelet aggregation resulting in thrombotic events, though this is rare. While significant abnormalities do occur, life-threatening bleeding complications of pit vipers in the US are very rare. 3. Neurotoxicity: Neurotoxicity is a less frequent but well-documented occurrence in specific areas of the country where local pit viper populations produce neurotoxic venoms. Symptom onset can be rapid. Most neurotoxins in the US result in a descending paralysis similar to botulism. Cranial nerves are typically affected first, followed by dysphagia and dysphonia and subsequently respiratory paralysis. In a small geographic region in the South East, ascending paralysis has been reported. 4. Myotoxicity: Local tissue damage, including damage to surrounding muscles, is common and may result in long-term disability. Rhabdomyolysis can occur in any pit viper envenomation but is more common from Timber Rattlesnake envenomation. 5. Rapid-onset shock and anaphylactoid reactions: This is a syndrome that develops over about five to 30 minutes consisting of hypotension, tachycardia, and shock along with various combinations of diarrhea, angioedema, rapid third-spacing of fluid with hemoconcentration. There is often minimal to no swelling at the bite site. The mechanism remains unclear. The leading theories include direct bradykinin effects or mast cell and basophil degranulation resulting in symptoms similar to anaphylaxis [11].
Plan the management of snake envenomations by type and severity	All patients who present with suspected Crotalidae envenomation should have labs and a physical exam to help distinguish dry or no bite from envenomation. Lab work should include CBC, platelets, PT, PTT, serum fibrinogen, CK, and UA. The affected extremity should be immobilized. The extremity should be elevated to reduce dependent edema that can cause significant pain that is in addition to the pain from the venom effect. Opiates and/or benzodiazepines can be utilized for symptom management, and NSAIDs should be avoided due to the platelet effect. Suction, ice, tourniquets, and empiric steroids (unless anaphylaxis) are not recommended. Patients with signs of envenomation: progression of edema, coagulopathy on lab work, or systemic signs of illness should be treated with antivenom [11].
Know which snake envenomations are liable to produce significant illness or injury in children	In addition to rattlesnakes, the pit vipers in the US include copperhead and cottonmouth snakes. These snakes share characteristics including a triangular head and elliptical pupils, heat-sensing pits, and two curved fangs. The other large family of venomous snakes in North America is the Elapidae family, which includes coral snakes. Envenomations by Eastern coral snakes can result in systemic neurotoxicity that includes paresthesias and bulbar symptoms that can progress to a respiratory compromise and paralysis. Texas coral snakes can also produce systemic neurotoxicity but are much more likely to cause significant local neuropathic pain in the affected extremity. Treatment includes supportive care, pain control, and observation. Coral snake antivenom production for a time was halted but has recently resumed production. It is available to treat Eastern and Texas coral snake envenomations [11].
Know the available antivenoms for pit viper envenomation and their indications, administration, and potential reactions	Crotalidae polyvalent immune Fab (ovine) (CroFab®) is manufactured from ovine Fab immunoglobulin fragments that are collected from the plasma of sheep who are immunized with the respective pit vipers venom. The venom-specific Fab fragment from the IgG molecule works by binding and neutralizing the toxin to remove the toxin from the tissues and is eliminated by the reticuloendothelial system [5]. Fab fragment antivenom has a quick onset of action of less than one hour with a half-life of approximately 40-60 hours [5]. The most important step is to establish IV access to obtain laboratory analyses and for prompt medication administration. It is important to obtain the labs prior to administering the antivenom. The next step is to delineate the severity of the patient to determine a dose of Crotalidae polyvalent immune fab. A patient with signs of envenomation is recommended to receive a loading dose of four to six vials. More severe envenomations may require eight to 12 vials as an initial loading dose. The dose is chosen based on clinical judgment. If initial control is not achieved within the first hour, the patient should receive additional doses of four to six vials continually as needed until control is obtained and reassessed hourly [3]. Examples of initial control would be local injury not progressing or enlarging, systemic symptoms being stabilized and/or abated, and/or the resolution of coagulopathies. Once it is determined that initial control is achieved, maintenance dosing can be considered. There is debate among snakebite experts as to if maintenance dosing is required. If maintenance dosing is recommended, the patient is to receive a maintenance dose of two vials every six hours for three more doses [3]. The patient should be monitored continuously for symptom resolution, infusion reaction, and allergic reaction. If at any time, progression of symptoms reoccurs, a loading dose should be initiated and the algorithm resets to step one. There are warnings and precautions that are associated with utilizing Crotalidae polyvalent immune Fab such as hypersensitivity reactions and infusion-related reactions. Approximately 0.6-6% of cases experience a hypersensitivity reaction ranging from very mild pruritus at the infusion site to severe including anaphylaxis. Hypersensitivity reactions can be acute or delayed reactions and therapy should be promptly discontinued followed by appropriate medical management. CroFab® contains papain, chymopapain, papaya extracts, and pineapple enzyme bromelain [3]. If the patient has a known allergy to any of these components, a risk/benefit discussion should be had with the patient, treating team, and consulting toxicologist. Infusion-related reactions can also occur and precautions are taken at the time of infusion. The initial infusion runs at a slower rate (25-50 mL/hr) for the first 10 minutes and then increases to complete the infusion over one hour. If the patient experiences an infusion-related reaction, the rate should be reduced to a rate that the patient tolerates and continues until therapy is complete. Despite appropriate treatment, delayed thrombocytopenia, typically 10-14 days after the envenomation. If coagulopathy returns, poison control should be contacted to decide if repeat doses of the medication are warranted. Crotalidae immune F(ab’)2 (equine), ANAVIP®, is manufactured from equine Fab immunoglobulin fragments that are collected from the plasma of horses who are immunized with the respective pit vipers venom [4]. ANAVIP® has the same mechanism of action as CroFab® where the venom-specific Fab fragment from the IgG molecule works by binding and neutralizing the toxin to remove the toxin from the tissues and is eliminated by the reticuloendothelial system [4]. There is limited data for pharmacokinetics of the antivenom; however, it is estimated to have a longer half-life than CroFab® of 150 hours with the same onset of action within one hour. ANAVIP® is unlike CroFab® in respect to dosing. Every patient will receive the same dose of 10 vials that is reconstituted in 250 mL of 0.9% normal saline. The final fluid volume can be adjusted for smaller pediatric patients if 250 mL over one hour would exceed recommended fluid resuscitation. If control is not obtained after the first dose, repeat doses of 10 vials as needed can be administered until control is achieved. Once initial control is obtained, if any symptoms return or if coagulopathy returns, four vials can be administered as needed [4]. Patients can develop an allergic reaction and caution should be taken with patients that have known allergies to horse protein. The infusion should be discontinued if any allergic reaction occurs followed by immediate medical management and poison control or a local toxicologist should be consulted. Infusion-related reactions are also a concern. It has the same infusion rate as CroFab®, slowly infusing over the first 10 minutes and then increased to complete infusion over one hour to ensure no infusion-related reactions occur.
Plan an appropriate disposition for the child bitten by a snake	Pit Viper “dry bites” may be discharged after eight to 12 hours if there are no signs or symptoms of envenomation, normal vital signs, normal initial and repeat laboratory work including coagulations studies, and if they are reliable to return for arranged follow-up. Patients who receive antivenom should be admitted to a closely monitored setting (ie ICU or capable SDU) for at least 18 hours following the antivenom doses [12].
Demonstrate strategies when a parent wants to leave against medical advice	When a parent would like to leave against medical advice or are declining an intervention, active listening is key. Understanding why the family does not want the therapy or why they want to leave may lead to productive discussions and elucidate misunderstandings and the family may then be on board with the plan. Asking open ended questions such as “Can you tell me why you are concerned/what you are concerned about?” allows guardians to voice their concerns and physicians to address them directly. Reviewing pros and cons of leaving against medical advice, including the risk of death, as well as asking another physician (if there is one available) to provide their opinion can be helpful in allowing families to understand the dangers of leaving against medical advice [13,14].
Know the process by which parental refusal for treatment can be overruled	Families may be reluctant to certain treatments and, rarely, ultimately choose to decline treatment and request to leave against medical advice. In the setting of a potentially life-saving intervention for a minor, such as anti-venom administration or blood product administration in the setting of hemorrhage, it is relatively straightforward in that the emergent treatment may be administered. In less extreme cases, for minor patients in which a therapy is not necessarily life saving, parents/guardians have a right to decline that treatment. If you are unfamiliar with a particular scenario in which care is being declined or if a family would like to leave against medical advice, we suggest reaching out to your hospital legal team. If, after utilizing the above discussed strategies to elucidate and allay concerns a family still would like to decline care, a good general rule is that if the minor patient’s life is immediately threatened by withholding the treatment, then the guardians’ refusal of treatment can be overruled. In some cases, medical custody may need to be pursued, which typically involves hospital legal as well as a judge granting this type of custody [11,12].
Recognize the concern related to health equity/lack of insurance and review resources available for uninsured/underinsured patient populations	Undocumented immigrants are not eligible to enroll in Medicaid or CHIP or to purchase coverage through the ACA marketplace. However, Medicaid payments for emergency services may be made on behalf of individuals who are otherwise eligible for Medicaid but for their immigration status. Additionally, some states have state-funded health programs that provide coverage to some groups of immigrants regardless of immigration status. [15-18].

Results

The scenario detailed in this technical report was piloted on several learner groups, including residents from Children’s Hospital of Philadelphia, Yale University, Seattle Children’s Hospital, and PEM fellows from the University of Washington. The scenario was facilitated both in-person as well as virtually, and Yale opted to include the health equity supplement while University of Washington included the anaphylaxis component. A total of 39 physician learners participated in the scenario and completed a survey reflecting their confidence with the learning objectives before and after simulation.

**Figure 3:**
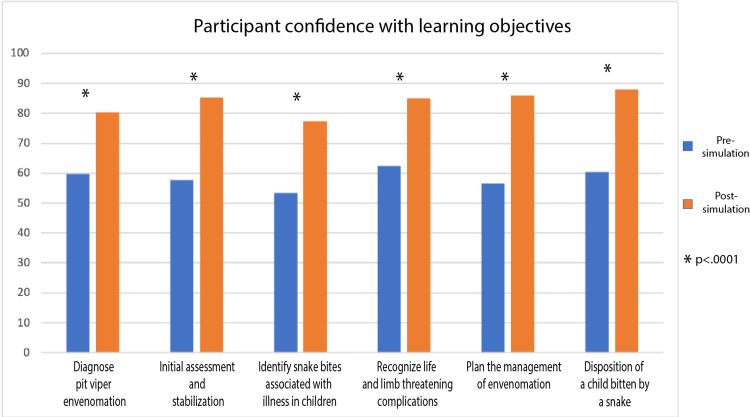
Participant Confidence With Learning Objectives

**Figure 4:**
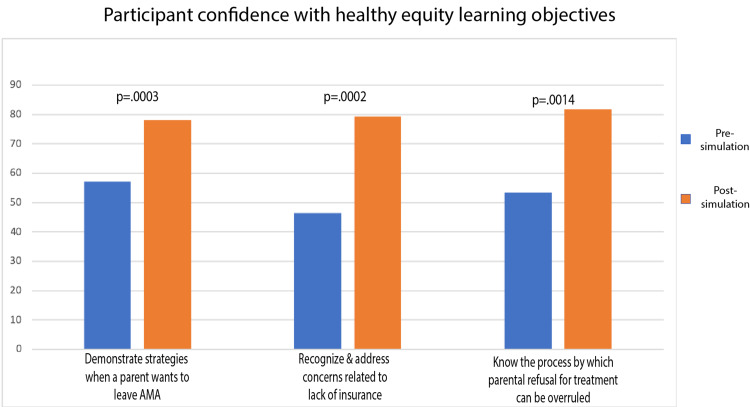
Participant Confidence With Health Equity Learning Objectives AMA: against medical advice

**Figure 5:**
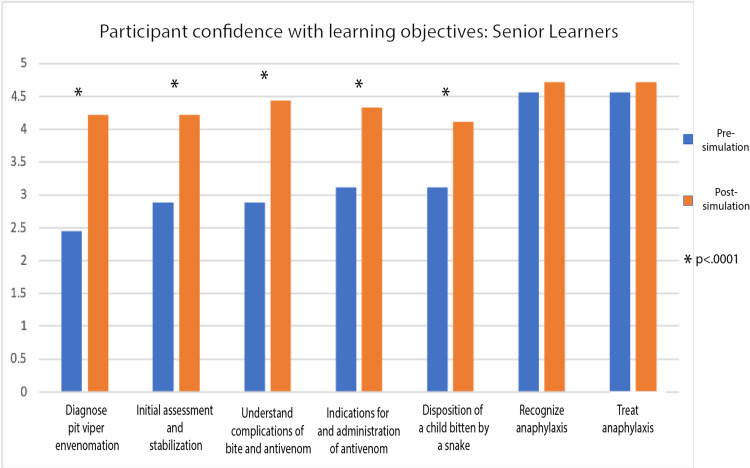
Participant Confidence With Learning Objectives: Senior Learners

For the core scenario there was a statistically significant increase in confidence for all of the learning objectives post-simulation (Figure 3). For those that completed the health equity supplement, there was also a statistically significant increase in confidence in the learning objectives related to parental refusal of treatment and resources for the uninsured (Figure 4). Finally, for the PEM fellow learners, we saw a statistically significant increase in confidence for all of the learning objectives except those related to anaphylaxis (Figure 5).

## Discussion

The intention of this scenario is that it can be tailored to the level and experience of the learner group as well as to a virtual or in-person platform and the learner responses support the efficacy of this approach. For learners without any prior experience, the case can be facilitated as a straightforward pediatric envenomation requiring antivenom with discussion focused on recognition and treatment of the disease process, communication with the team, family, and consultants, resource utilization, and appropriate dispositioning. For those with some experience with toxicology or envenomations, such as senior residents, PEM, or wilderness medicine fellows, the presentation can be augmented to review the core concepts of pit viper management and introduce considerations regarding the cost of care, parental rights of refusal, and federal as well as institution-specific resources available. These health equity components are an important part of this patient presentation given the cost of antivenom and subsequent ICU stay and are also applicable to a number of presentations to the pediatric ED.

With the inclusion of the health equity component, the simulation and debrief do require more allotted time than a typical simulation scenario. To address this, the authors utilized a "pause and restart" approach to the case, where the simulation was paused after the health equity portion and was debriefed with the aid of a social worker and content expert. Once that debrief was sufficiently concluded, the simulation was resumed with the administration of antivenom and carried through disposition. The teamwork, communication, and medical management of that portion of the scenario were then debriefed. This divided the information into manageable portions and, overall, the scenario was completed in what would have been the allotted time for two back-to-back simulation cases.

The medical management aspects of the scenario can also be tailored to a more senior group of learners, such as PEM fellows as was done in our case. Participants in the simulated scenario reported increased confidence in all learning objectives with the exception of recognizing and treating anaphylaxis in the senior learner group. The lack of change in confidence with the anaphylaxis learning objectives in this senior learner group likely reflects the existing high comfort level of PEM fellows with anaphylaxis as a disease process, as evidenced by the pre-simulation score of greater than 4.5/5. We feel that the anaphylaxis component is still important to include either as part of the simulated patient presentation or during the subsequent debrief as it orients learners to be aware of this potential complication with antivenom treatment in pit viper envenomation as well as the steps necessary to stabilize the patient while continuing to treat the envenomation.

Finally, we include an option for virtual facilitation of this case. The VRR platform allows for increased interactivity and task completion fidelity while facilitating a virtual scenario as may be necessary for coronavirus disease (COVID) restriction compliance and/or distance learning objectives. The VRR hosted website has an included orientation video, which we found accessible and effective in preparing our learners for the simulation. The ability for both facilitators and learners to interact with the virtual document provided for increased participant interaction as opposed to strictly zoom-based scenarios. The authors also found that the existing platform allowed for decreased preparation time for facilitation of the scenario and more time to customize the information and media components.

## Conclusions

Pit viper envenomation is a rare but important cause of presentations to the ED. This scenario details the presentation of a child who has been bitten and reviews the presentation, initial stabilization, workup, treatment, and disposition of the envenomated child. We also include an optional health equity supplement to address insurance and parental refusal concerns as well as an optional inclusion of anaphylaxis to the antivenom for more senior learners. This scenario can be run in-person or virtually, and we include a resource for virtual facilitation.

## Appendices

Appendix A:

Health coverage of undocumented immigrants

In 2018, there were 22 million noncitizens living in the US who accounted for 7% of the total population. Four in 10 of the noncitizens were undocumented immigrants. Many children live in mixed immigration status families that may include lawfully present immigrants, undocumented immigrants, and/or citizens. One in four children has an immigrant parent and the majority of these children are citizens.

Undocumented immigrants are not eligible to enroll in Medicaid or Children’s Health Insurance Program (CHIP) or to purchase coverage through the Affordable Care Act (ACA) marketplace.  However, Medicaid payments for emergency services may be made on behalf of individuals who are otherwise eligible for Medicaid but for their immigration status. These payments can cover costs for the emergency care of undocumented immigrants. Since 2002, states have had the option to provide prenatal care to women regardless of immigration status by extending CHIP coverage to the unborn child. In addition, some states have state-funded health programs that provide coverage to some groups of immigrants regardless of immigration status. There are also some locally-funded programs that provide coverage or assistance without regard to immigration status. Locally, social workers, legal aid organizations, the office of the healthcare advocate, and community-based organizations may be additional resources to help families with health insurance and/or immigrant rights.

Appendix B:

Discharge against medical advice and children

Discharge against medical advice (DAMA) refers to instances in which patients are discharged from a health care setting against the recommendation of their clinician. With children, this can be due to the wishes of the substitute decision-maker (SDM), often a parent. Reasons for DAMA in children included a caregiver’s perception that the child is well, financial constraints, inconveniences due to a hospitalization, dissatisfaction with the child’s care, hopelessness about the clinical situation, or preferences for traditional forms of treatment.

When clinicians are faced with DAMA requests for children, they must ensure the child’s best interests are met, understand their professional obligations, and engage in guided discussions with families that include shared and informed decision-making strategies. State laws and hospital policies will provide clinicians with guidance regarding what it means to act in a child’s best interests and guide clinicians about when to engage in child protection services. Once the capacity of the older child or caregiver is established, clinicians must try to understand why the caregiver may want to leave, their concerns, and whether they recognize the risks of leaving. They should also assess the caregiver’s understanding of the medical status and treatment of the child. Finally, they should discuss any other reasonable alternatives. Ultimately, the guidance from the law states the primary consideration on whether to proceed with DAMA has to be the child’s health and well-being, and if the clinician thinks that DAMA will cause harm to the child, then the clinician is legally and ethically obliged to involve child protection services. However, if the child’s best interests have been met (even if it may not be the action the clinician might recommend the most), clinicians may move forward with DAMA. If proceeding with DAMA, assure documentation of parental competence, the options discussed including the potential outcomes of the DAMA, and the parental signature.
